# The diagnosis of fetal micrognathia with two-dimensional and real-time three-dimensional color Doppler ultrasound

**DOI:** 10.4314/ahs.v25i2.7

**Published:** 2025-06

**Authors:** Jing Zhong, Zeyu Deng, Xinyue Yang

**Affiliations:** Department of Ultrasound, Xiangyang No.1 People's Hospital, Hubei University of Medicine, Xiangyang 441000, China

**Keywords:** fetal micrognathia, Two-dimensional, three-dimensional, color Doppler ultrasound

## Abstract

**Background:**

The study describes the diagnose of fetal micrognathia by the two-dimensional and real-time three-dimensional color doppler ultrasound, and evaluates its clinical value.

**Methodology:**

The case group comprised 100 fetuses with highly suspected mandibular deformity, and the control group consisted of 100 normal fetuses. Various ultrasonic equipments and multimedia data processing system were used to obtain 2D and 3D images of fetal mandible, and the Biparietal Diameter (BPD) and Fetal Mandibular Longitudinal Diameter (FML) were measured. The Jaw Indices (JI) and Inferior Facial Angle (IFA) were calculated, and all suspected cases underwent chromosome examination. Eight confirmed cases of mandibular deformity were identified in the case group.

**Results:**

This study examined eight cases of fetal micrognathia and found positive correlations between transverse diameters and FMLs of normal fetuses and gestational weeks. There was no significant correlation between JI, IFA, and gestational weeks. The study also compared JI, IFA, and FML between normal and small mandibular groups, finding significant differences. The smallest FML in the small mandibular group was only approximately equal to the normal fetus FML in the 18th week.

**Conclusion:**

The study provides insights into the measurement and analysis of fetal micrognathia. Combining FML, JI, and IFA with ultrasonic diagnosis reduces fetal micrognathia misdiagnosis.

## Introduction

Usually accompanied by various chromosomal abnormalities, fetal micrognathia can coexist with structural abnormalities in multiple parts. Due to the severe retraction or loss of the mandible, the neonates tongue would hamper the space between the maxilla and the oral cavity, cause neonatal respiratory distress syndrome and asphyxia, and even can cause neonatal death-affect the perinatal survival rate, so prenatal ultrasonographic diagnosis of fetal micrognathia has a very important clinical significance. Previously the ultrasonic diagnosis of fetal micrognathia mainly depended on the subjective judgment of sonographers on fetal coronal imaging. There are still a few scientific ultrasonic methods and standards for normative measurement of mandibular length and mandibular facial angle to evaluate fetal micrognathia. The study describes the diagnose of fetal micrognathia by the two-dimensional and real-time three-dimensional color doppler ultrasound and evaluates its clinical value.

## Materials and methods

### Research objects

The fetuses mentioned in the study all signed relevant ethical agreements.

The case group consisted of 100 fetuses with highly suspected mandibular deformity after visual coronal and sagittal facial imaging during prenatal ultrasound examination in our hospital from January 2021 to December 2022. Among the highly suspected cases, 8 cases of mandibular deformity were finally confirmed by induced labor. The control group consisted of 100 normal fetuses who underwent prenatal ultrasonography in our hospital from January 2021 to December 2022. Inclusion criteria of case group and control group: singleton, the fetuses in a good condition, 18∼32 weeks gestational age, pregnant women of 18∼32 years old, aged 22±4.9 on average. Exclusion criteria: six deformities by the Ministry of Health (anencephaly, spina bifida, fatal achondroplasia, encephalocele, viscera bareness, single chamber heart), gestational hypertension, gestational diabetes, and with other pre-existing conditions.

### Instruments and methods

Used ultrasound equipments were a GE-E8 with an AC2-5 dopper probe (3.5 MHz), a RAB2-5L three-dimensional probe (5.5 MHz), and a SIEMENS-ACUSON S2000 with a 4C1 dopper probe (4.5 MHz), a 7CF2 three-dimensional probe (5.5 MHz). In the meantime, the ultrasonic graphic multimedia data processing system was necessary. When the fetal position was appropriate, the fetal mandibular imaging should be carefully observed, two-dimensional ultrasound images of fetal coronal plane, sagittal plane and transverse plane were taken and saved. The Biparietal Diameter (BPD) of fetal head was measured in the standard thalamus plane. The fetal was showed mandible completely and symmetrically on the basis of thalamic plane when the probe was rotated clockwise 30∼45°. The transverse diameter was the widest diameter of bilateral mandible; The Fetal Mandibular Longitudinal Diameter (FML) was the vertical distance from the midpoint of fetal mandible trasverse diameter to the fetal median mental junction. The Jaw Indices (JI) were calculated (JI=FML/BPD×100%). The JI of a suspected case was below 50%. The fetal forehead, nasal bone, nasal tip, upper lip, lower lip and lower jaw were displayed in the sagittal plane of the fetus and the shape of the upper lip, lower lip and chin curve were carefully observed, and then the fetal mandibular facial angle was measured. The specific method was: a straight line was taken from the highest point of the nasal bone that was perpendicular to the prefrontal bone; another line was taken from the styloid process of the left and right mandible, namely the lowest point of the chin, to the most prominent upper/lower lip. The angle value of the intersection of the two lines was the Inferior Facial Angle (IFA). An IFA < 50% case was regarded as a suspected case, all parameters were measured for three times, then averaged the results. With the 3D color ultrasound real-time imaging technology, we captured the faces of the suspected group fetus in multiple sections, observed the jaw, chin, lips and ears, and checked if there were other malformations or not. All fetuses in the suspected group underwent chromosome examination.

### Statistical analysis

With the help of the Statistical Product and Service Solutions (SPSS) 17.0 software (SPSS Inc., Chicago, IL, USA), comparison of mean values between the small mandibular group and the normal control group had adopted two independent samples t-test. Measurement data are expressed by mean differences ± standard deviation (x̅ ± s); The relationships between normal fetal FML- transversal diameter-JI, IFA and gestational age were analyzed by Person correlation analysis. P values < 0.05 were considered statistically significant.

## Results

8 cases of fetal micrognathia were confirmed via ultrasonic measurement of fetal IFA, FML, transverse diameter and JI analysis (Table 1). Among these cases, 2 were combined with hydramnios, 1 were combined with 18-Trisomy Syndrome, 1 was combined with Pierre Robin syndrome, 1 was combined with ventricular septal defect, 1 was combined with omphalocele, 1 was combined with Treacher-Collins syndrome, and 1 rare case was combined with multiple facial deformities (serrated pumpkin cleft lip, cyclopia, single nostril, flat nose, low ear position, external auditory canal structural abnormality). There was a positive correlation between the transverse diameters and FMLs of normal fetuses and their gestational weeks (Chart.1, the scatter diagram, R was 0.952 and 0.954 respectively, all P<0.01), and there was no significant correlation between JI, IFA and gestational weeks.

**Table 1:** JI, IFA and FML of 8 cases of fetal micrognathia

Gestational age	Number	JI(%)	IFA(°)	FML(mm)
18-24	3	29.30±1.67	37.33±0.58	16.90±1.12
25-28	3	25.23±0.84	37.33±3.21	18.33±0.68
29-32	2	25.55±0.49	38.50±4.95	19.55±0.91

Table 2 showed the mean values of the transverse diameters, FMLs, JIs and IFAs of normal fetuses at 18-32 gestational weeks in this region.

**Table 2:** Mandible related measurements of normal fetuses at 18-32 gestational weeks in this region

Gestational age	Number	Transverse diameters(mm)	FML(mm)	JI(%)	IFA(°)
18-24	59	32.95±1.72	29.36±1.35	49.68±2.30	58.92±2.85
25-28	23	37.96±1.99	35.97±1.83	50.55±2.40	58.09±3.30
29-32	10	41.99±1.92	36.87±1.37	51.33±1.95	58.70±2.98

The comparison results of JI and IFA between the normal group and the small mandibular group (Table 3) show that, IFAs in the small mandibular group were all < 45°, IFAs in the normal group were ranged from 51° to 63°; JI in the small mandibular group were ranged from 35% to 42%, the normal group JI was all approximately equal to 50%. JI and IFA in the small mandibular group were significantly lower than those in the normal group, and the difference was statistically significant.

**Table 3:** The comparison results of JI and IFA between the normal group and the small mandibular group

Parameter	Normal group	Small mandibular group	P
JI(%)	50.08±2.34	26.84±2.28	<0.01
IFA(°)	58.68±2.97	37.63±2.62	<0.01

The comparison results of FML between the normal group and the small mandibular group (Table 4) show that, FML of normal fetus in the control group was positively correlated with gestational age and the average value of 59 fetuses was about (29.36±1.35) mm from the 18^th^ to 24^th^ week, (35.97± 1.83) mm from the 25th to 28th week, (36.87±1.37) mm from the 29^th^ to 32^th^ week. As for the small mandibular group, the lowest FML was about 16mm in the 18^th^ week, 18 mm the 28^th^ week, and 20 mm the 32^th^ week that was only approximately equal to the normal fetus FML in the 18^th^ week.

**Table 4:** The comparison results of FML between the normal group and the small mandibular group

Gestational age	Normal group	Small mandibular group	P
18-24	29.36±1.35	16.90±1.12	<0.01
25-28	35.97±1.83	18.33±0.68	<0.01
29-32	36.87±1.37	19.95±0.35	<0.01

## Discussion

Prenatal ultrasound is a safe and noninvasive medical examination method and widely used in fetal malformation screening around the world. It can accurately diagnose to most fetal malformations before birth, and has irreplaceable important clinical value in reducing birth defects, improving population quality and newborn survival^1^.

Fetal micrognathia mainly refers to short mandible or no mandible^2^,^3^, and its pathological causes may be chromosome deformity, bone dysplasia, gene syndrome, etc^4^,^5^. Some scholars believe that the mandible is formed by the development of the abnormal branchial arch development that can cause abnormalities in the mandible, maxilla and ear. Fetal micrognathia is mostly caused by the damage of the blood supply artery of the parotid arch, which affects its development^6^,^7^. Most of fetal micrognathia had chromosomal abnormalities, which can also be found in many fetal chromosomal malformation syndromes. In this study, 72% of the suspected cases had chromosomal abnormalities. Scholars confirmed that 60 to 70 percent of fetal micrognathia had chromosome abnormalities, especially do the 18-Trisomy and 13-Trisomy^8^,^9^. Among the 8 fetal micrognathia cases diagnosed in our hospital, 1 case had 18-Trisomy. Other structural abnormalities are often associated with fetal micrognathia. One rare case in our research was combined with multiple facial deformities (serrated pumpkin cleft lip, cyclopia, single nostril, flat nose, low ear position, external auditory canal structural abnormality). According to the past diagnosis experience, almost cyclopia and single nostril were found in holoprosencephaly. However, there was no obvious abnormality in the intracranial structure of this fetus. Due to the fact that many fetal micrognathia had chromosomal abnormalities, it has a poor prognosis and in severe cases, the survival of newborns would be affected.

Most of the previous ultrasonic diagnosis methods relied on visual observation and lacked objective data. And for early or mild to moderate fetal micrognathia, the false negative rate and misdiagnosis rate are on high levels. The three ultrasonic data indicators (FML, JI, IFA) in this study combined with 3D color ultrasound real-time imaging technology have important clinical application value for prenatal diagnosis of fetal micrognathia, which can reduce birth defects and improve population quality^10^. Two-dimensional and three-dimensional ultrasound imaging of fetal micrognathia: the sagittal plane of the fetus shows abnormal facial curve on the sagittal plane. There are four hump signs (nose tip, upper lip, lower lip and lower jaw) in sagittal plane of normal fetus, and the tops of the three hump signs are basically located at a horizontal line except the nose tip^11^,^12^. The mandible of the micrognathia fetus moves backward or disappears, which is significantly lower than the hump level line. And the lower lip also moves backward due to jaw retraction. The typical features are “prominent upper lip and small chin”. During the whole examination, the fetus is in an open-mouth status. In severe cases, the fetal tongue can be seen extending out of the mouth and its movement can be seen. The chin is very small or not developed of the micrognathia, and the continuous smooth curve of the normal cheek and jaw is interrupted at the corners of the mouth on both sides in coronal plane. The three-dimensional imaging more intuitively and vividly shows that the obviously shortened mandible, the small chin, the open-mouth status and the extended tongue, and the severe case may occur that the chin would be spot like echo or not be developed.

### Ultrasonographic Method of fetal micrognathia

FML: FML was measured after the mandible was showed from the thalamic plane. FML of normal fetus in the control group was positively correlated with gestational age and the average value of 500 fetuses was about (23.09 ± 2.07) mm from the18th to 24^th^ week, (32.61±0.28) mm from the 25^th^ to 28^th^ week, (37.28±0.83) mm from the 29^th^ to 32th week. As for the small mandibular group, the lowest FML was about 12mm in the 18th week, 16 mm the 28^th^ week, and 19 mm the 32^th^ week that was only approximately equal to the normal fetus FML in the 18th week. It is suggested that the FML value of the small mandibular group is significantly smaller than that of the normal fetus at the same gestational week.

JI: JI=FML/BPD×100%. In normal fetus, FML is about half of BPD^13^,^14^. JI of 500 normal fetuses in the control group all follow this rule while the value in the study group was significantly less than 50%. JI in the small mandibular group were ranged from 22.3% to 39.4%. The difference between the two groups was statistically significant.

IFA: IFAs in the small mandibular group were all < 45°, IFAs in the normal group were ranged from 55°to 69°. We investigators believe that two-dimensional ultrasound imaging can clearly show the skeletal structure of the fetal face, which is better than three-dimensional imaging to measure IFA. The retractions of fetal mandible within the normal range can occur when IFA were 45°to 55°, resulting in false positive results. Two fetuses in study group were suspected to have micrognathia by IFA measurement, and were finally diagnosed as mandibular retraction by FML and JI measurement. The reasons may be that the upper lip of the fetus is more prominent in the sagittal plane, and IFA measurement would be affected when the sagittal display is limited due to the fetal limbs shade the face^15^,^16^. All these above could cause the IFA measurement be lower than the normal value, resulting in the false micrognathia image. Mandibular retraction within the normal range will not produce clinical symptoms of fetal micrognathia and will not affect the survival of this fetus. Therefore, when IFA is slightly lower than the normal value, it should be further diagnosed with caution in combination with FML and JI measurement.

From the screening and diagnosis o of 8 cases of mandibular deformities, we found that FML and JI were the first choice for the diagnosis of fetal micrognathia.

In this study, we concluded that FML (significantly lower than the normal value of the same gestational week), JI<31% and IFA<45% were the comprehensive ultrasonic data indicators for prenatal diagnosis of fetal micrognathia. The application of FML, JI and IFA combined with traditional ultrasonic diagnosis can reduce the misdiagnosis and miss-diagnosis of fetal micrognathia, which has important clinical significance for reducing birth defects and improving population quality.
